# Outcome after hepatic resection for isolated non-colorectal, non-neuroendocrine liver metastases in 100 patients – the role of the embryologic origin of the primary tumor

**DOI:** 10.1186/s12893-018-0424-1

**Published:** 2018-10-29

**Authors:** Philipp Anton Holzner, Frank Makowiec, Andrea Klock, Torben Glatz, Stefan Fichtner-Feigl, Sven Arke Lang, Hannes Philipp Neeff

**Affiliations:** Department of General and Visceral Surgery, Center for Surgery, Medical Center – University of Freiburg, Faculty of Medicine, University of Freiburg, Hugstetterstr. 55, D-79106 Freiburg, Germany

**Keywords:** Non-colorectal, Non-neuroendocrine, Liver metastases, Hepatic resection, Surgery, Survival, Outcome, Risk factor

## Abstract

**Background:**

The indication for hepatic resection (HR) in patients suffering from liver metastases (LM) other than colorectal and neuroendocrine tumors is one focus of current multidisciplinary, oncologic considerations. This study retrospectively analyzes outcome after HR for non-colorectal, non-neuroendocrine (NCNNE) LM in the absence of distant or extrahepatic metastases.

**Methods:**

We included 100 consecutive patients undergoing HR for isolated NCNNE LM from a prospective database in our institution, including postoperative follow-up. Primary tumors were of mesodermal origin in 44%, of ectodermal origin in 29% and of entodermal origin in 27%. Survival analysis was performed by univariate and multivariable methods. Mean follow-up after hepatic surgery was 3.6 years (0.25–16).

**Results:**

Median age at the time of HR was 59.5 years. Kaplan-Meier-estimated survival after liver resection was 56.8%, 34.3% and 24.5% after 5, 10 and 15 years, respectively. Univariate analysis after HR revealed residual disease (hepatic or primary; *p* = 0.02), female gender (*p* = 0.013), entodermal origin (*p* = 0.009) and early onset of metastatic disease (≤24 months, *p* = 0.002), as negative prognostic factors. Multivariable survival analysis confirmed residual disease, female gender, entodermal embryologic origin and early onset of metastatic disease (≤24 months) as independent negative prognostic factors.

**Conclusion:**

Overall outcome after HR of NCNNE LM results in acceptable long-term outcome. Although individual decision-making today mostly relies on clinical experience for this type of disease, risk factors derived from the embryologic origin of the tumor might help in patient selection.

## Background

Hepatic resection has become a standardized, well-established surgical procedure with low morbidity and mortality. Owing to growing experience in hepatobiliary surgery, better understanding of tumor biology, effective pharmaco- and other multidisciplinary therapies, the therapeutic approach in metastatic colorectal carcinoma (mCRC) has completely changed during the last two decades [1–3].

In CRC, large studies elucidated a survival benefit after liver resection for hepatic metastases with a 5-year overall survival of up to 74% [3]. Current evidence shows a benefit for resection of hepatic metastases in CRC independent of number or size, as long as complete tumor clearance can be accomplished [4]. Likewise, surgical treatment of metastases from neuroendocrine tumors (NET) has emerged over recent years [5, 6]. In contrast to CRC, cytoreductive surgery for symptomatic hormone-active metastases has also become an accepted treatment for NET [7–9].

In addition, surgically-unresectable NET LM are frequently treated by other local approaches such as arterial embolization, thermo ablation, selective internal radiotherapy (SIRT) and liver transplantation [10].

As a consequence, a growing number of well-selected patients undergo hepatic resection for NCNNE LM, preferably after discussion in interdisciplinary tumor boards [11]. However, substantial evidence for the benefit of such surgical treatment is still unavailable, partially due to the relatively small number of patients being resected with NCNNE LM compared to CRC or NET. Also, an inherent heterogeneity of the arbitrarily defined NCNNE groups has most probably led to the conflicting results available today from case series [11–14]. Selecting surgical candidates for NCNNE LM resection at present is based more on clinical decision- making for individual patients following surgical criteria than on oncological selection criteria.

While various risk factors and also prognostic factors have been identified [11, 12, 15–17], none of those factors could be unanimously reproduced by different centers.

The aim of the present study was to describe estimated Kaplan-Meier survival after HR for NCNNE liver metastases only and to identify prognostic factors for long-term survival in 100 patients after HR in a single academic hepatobiliary surgical center.

## Methods

### Criteria for study inclusion

**Fig. 1:**
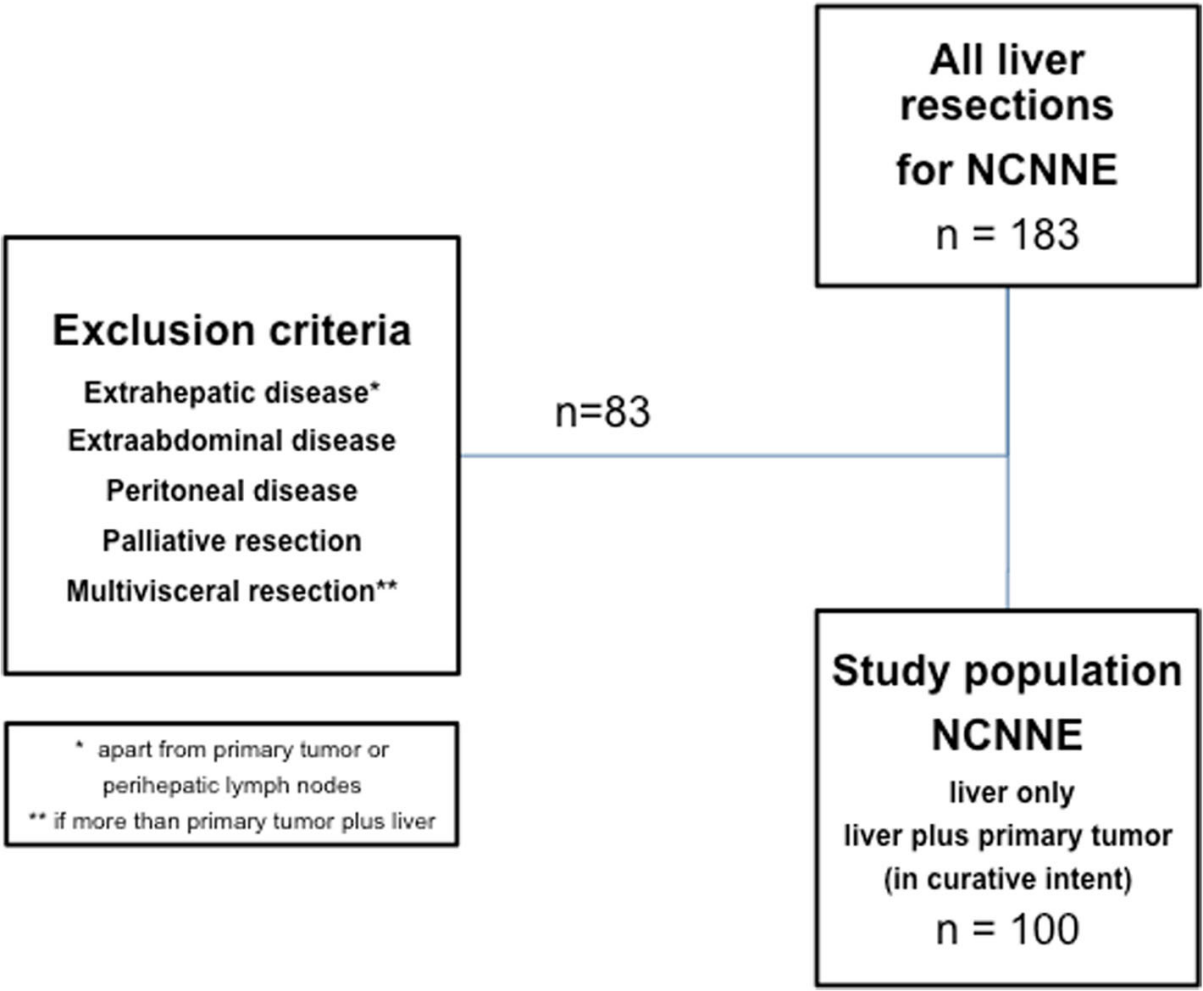
Patient selection from database 1999-2015

Our prospective liver database was analyzed from first HR in NCNNE metastases performed from 1999 until 2015. In order to achieve a uniform data set, distinct exclusion criteria were applied: Patients with extrahepatic disease (EHD) (except synchronous primary tumor specimen or lymph node involvement in the hepatoduodenal ligament), extra-abdominal tumor manifestation, patients who underwent surgery for palliative causes and incomplete (prior) resection of the primary or other widespread extrahepatic tumor (resulting in R2 resections/debulking) were excluded from this analysis. Concomitant peritonectomy and multivisceral resections were also excluded from further analysis. Patients who had simultaneous resection of liver metastases and the primary tumor, however, were included in this study (Fig. 1).

Lymph node involvement extending to the hepatoduodenal ligament was considered as local metastatic disease as long as affection was restricted to lymph nodes number 12 a, 12 b and 12p according to the Japanese classification [18] and these patients were also included. Direct organ involvement by liver metastases per continuitatem (i.e. contact with diaphragm, colon, omentum etc.) was not defined as extrahepatic disease (EHD) and therefore not considered as an exclusion criterion, as long as complete resection was achieved.

### Surgical technique

All HR were performed by one team of experienced hepatobiliary surgeons. A Cavitron Ultrasonic Surgical Aspirator (CUSA; Plainsboro, New Jersey, USA) in conjunction with bipolar (irrigated) forceps was used in all resections. Hepatic and overall resection margins were defined to be “negative” (R0) if at least “one cell layer” of healthy tissue was present at the specimen (submillimeter definition).

### Subgroups and definitions

For further analysis, cases were grouped according to clinical, pathological and also embryological aspects of the primary tumor. Factors were also retrieved to calculate a score proposed by Adam et al. [11]. This score is derived from a retrospective pooled multicenter data analysis and consists of 0–10 points. Factors included are extrahepatic metastases (1 point), major hepatectomy (1 point), R2 resection (1 point), age (max. 2 points), disease-free interval between treatment of primary (max. 2 points), primary tumor histology (max. 3 points).

### Data acquisition and statistics

All patient-related perioperative data were extracted from our prospectively- maintained hepatic surgery database. Further data were retrieved from the electronic patient charts at our institution. Survival status was continuously obtained at 3- month intervals from the institutional registry at our comprehensive cancer center which is based on the local authority registration office and/or from the computerized hospital information system (e.g. if death occurred in-hospital). Estimated survival from the time of first HR was analyzed using the Kaplan–Meier method, followed by a log-rank test for the comparison of subgroups. Multivariable survival analysis was performed with the Cox proportional hazard model (forward selection strategy using a likelihood ratio statistic; inclusion *p*-value = 0.1) including the report of relative risks (RR) and their 95% confidence interval. All data analyses were performed using SPSS (IBM SPSS Statistics for Windows, Version 23.0. Armonk, NY; IBM Corp.).

## Results

### Study population

Between 1999 and 2015, 183 patients underwent HR for NCNNE LM in our institution. A total of 100 patients met the inclusion criteria and were included in the final analysis (Fig. 1).

### Demographics

The median age at the time of surgery was 59.5 years (range 26–79). Half of the patients were aged 30–60 (50%), and 49 patients (49%) were older than 60 years. Only one patient was younger than 30 years. Women represented 60% of patients.

Mean follow-up after hepatic surgery was 3.6 years (0.25–16).

Histology of the resected liver metastasis showed 58% adenocarcinomas, 14% melanomas, 12% gastrointestinal stroma tumors (GIST), 12% (soft tissue) sarcomas, 1% squamous cell carcinomas and 3%, with primary histology missing. Classification was based on the site of previous surgical tumor removal and the histology of the removed liver lesion, therefore termed “ill-defined”). Mesodermal, ectodermal and entodermal origin was found in 44, 29 and 27%, respectively. 3 “ill-defined” primary tumors (with missing primary histology) were placed in the entodermal group based on the site of surgical primary removal. The specific primary tumor sites (regardless of histological appearance) are given in Table 1.

**Table 1:** Specific tumor origin in 100 patients

Embryologic Origin	Tumor Origin	*n* = 100
Mesodermal		44
	GIST	12
	Ovarian	11
	Renal	8
	Soft tissue sarcoma	6
	Uterine	3
	Testicle	2
	Fallopian Tube	1
	Hemangiopericytoma	1
Ectodermal		29
	Breast	15
	Cutaneous Melanoma	11
	Uveal Melanoma	3
Entodermal		27
	Pancreatic	7
	Gastric	5
	Duodenal (ampullary)	3
	Ill defined primary^a^	3
	Esophagus	2
	Prostate	2
	Small bowel	2
	Extrahepatic Bile Duct	1
	Thyroid	1
	Lung	1

^a^primary histology missing, classification was based on the site of previous surgical tumor removal and the histology of the removed liver lesion

### Univariate survival

**Fig. 2:**
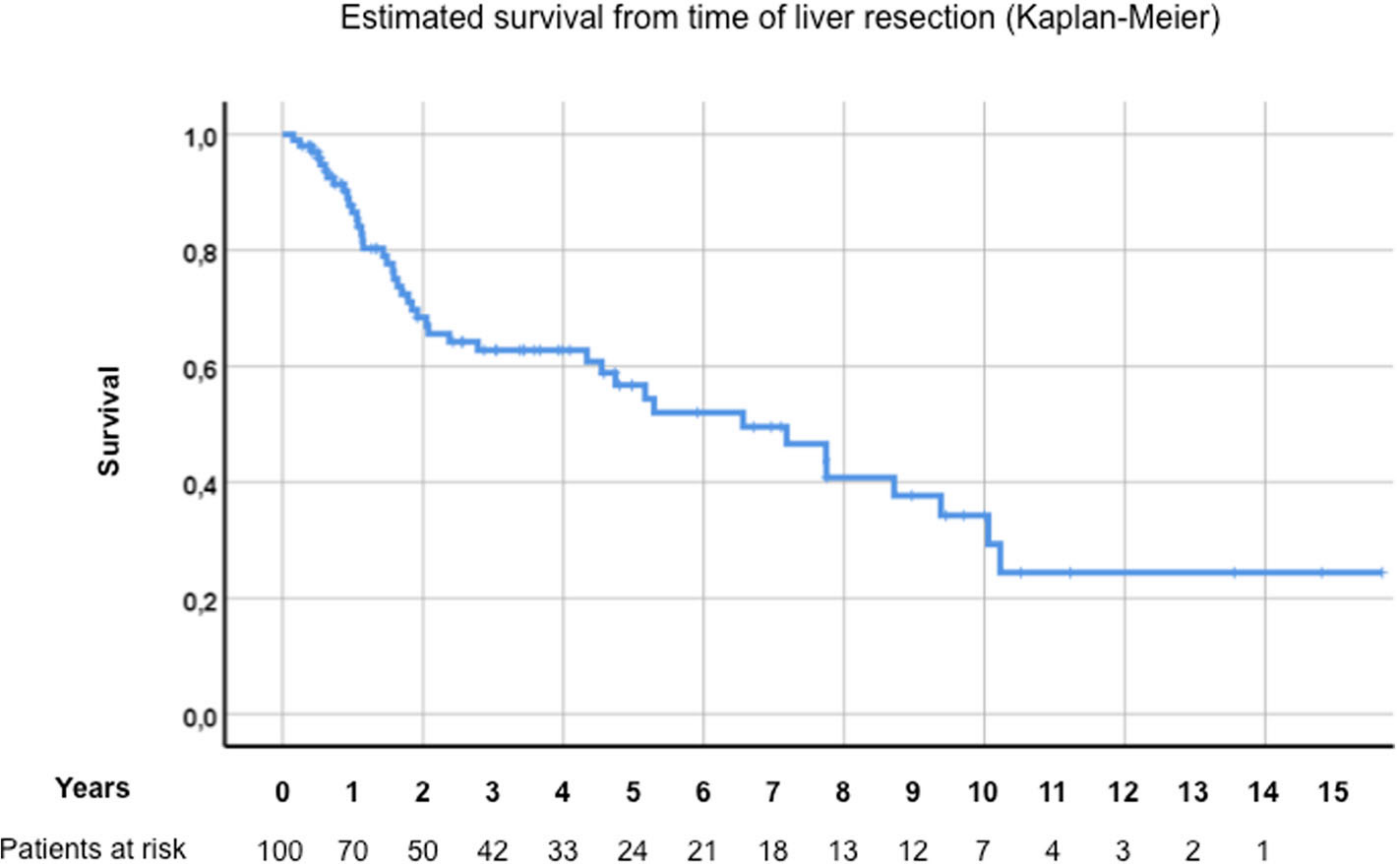
Overall Survival

**Fig. 3:**
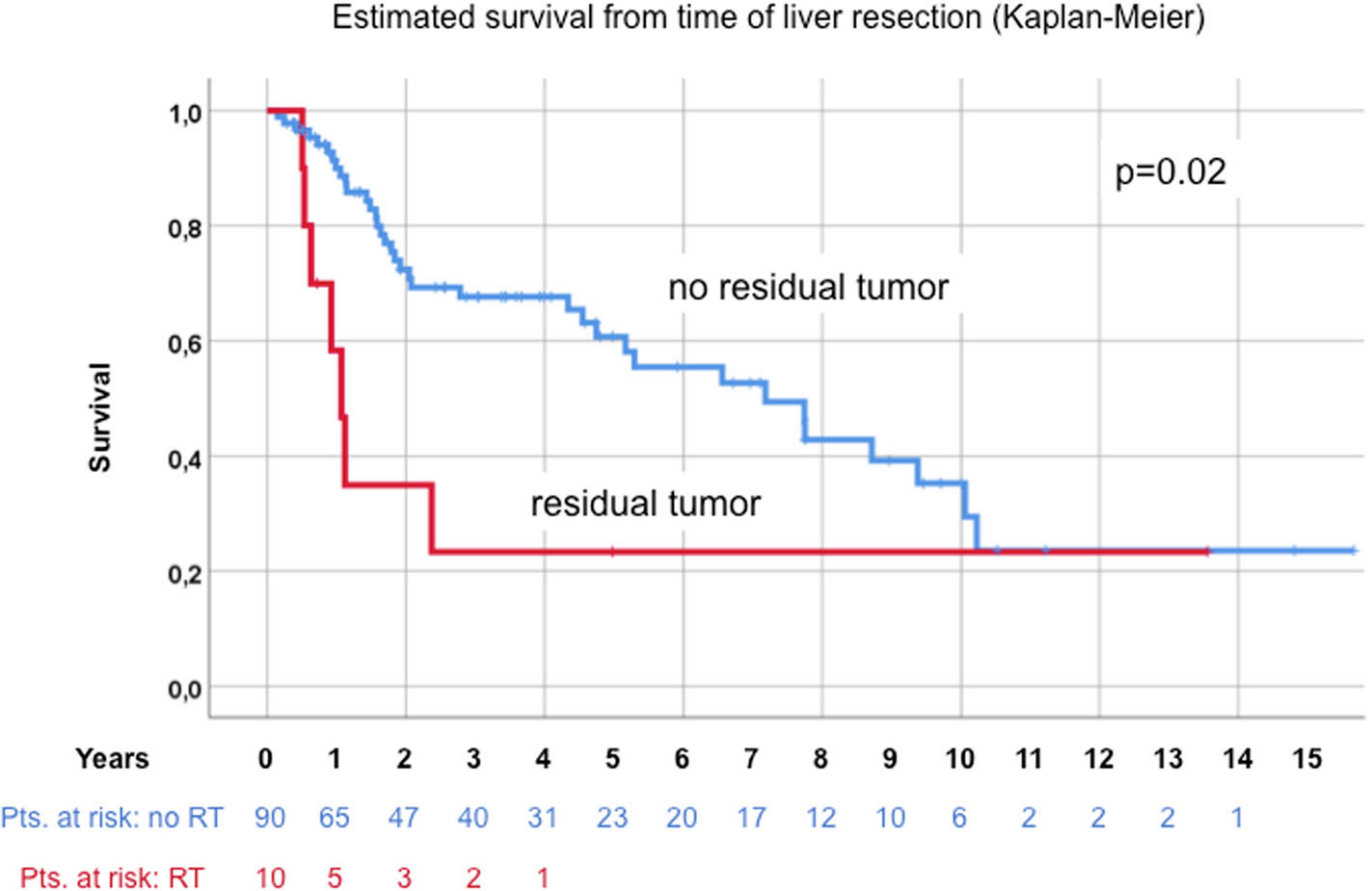
Resection status no residual vs. residual tumor

**Fig. 4:**
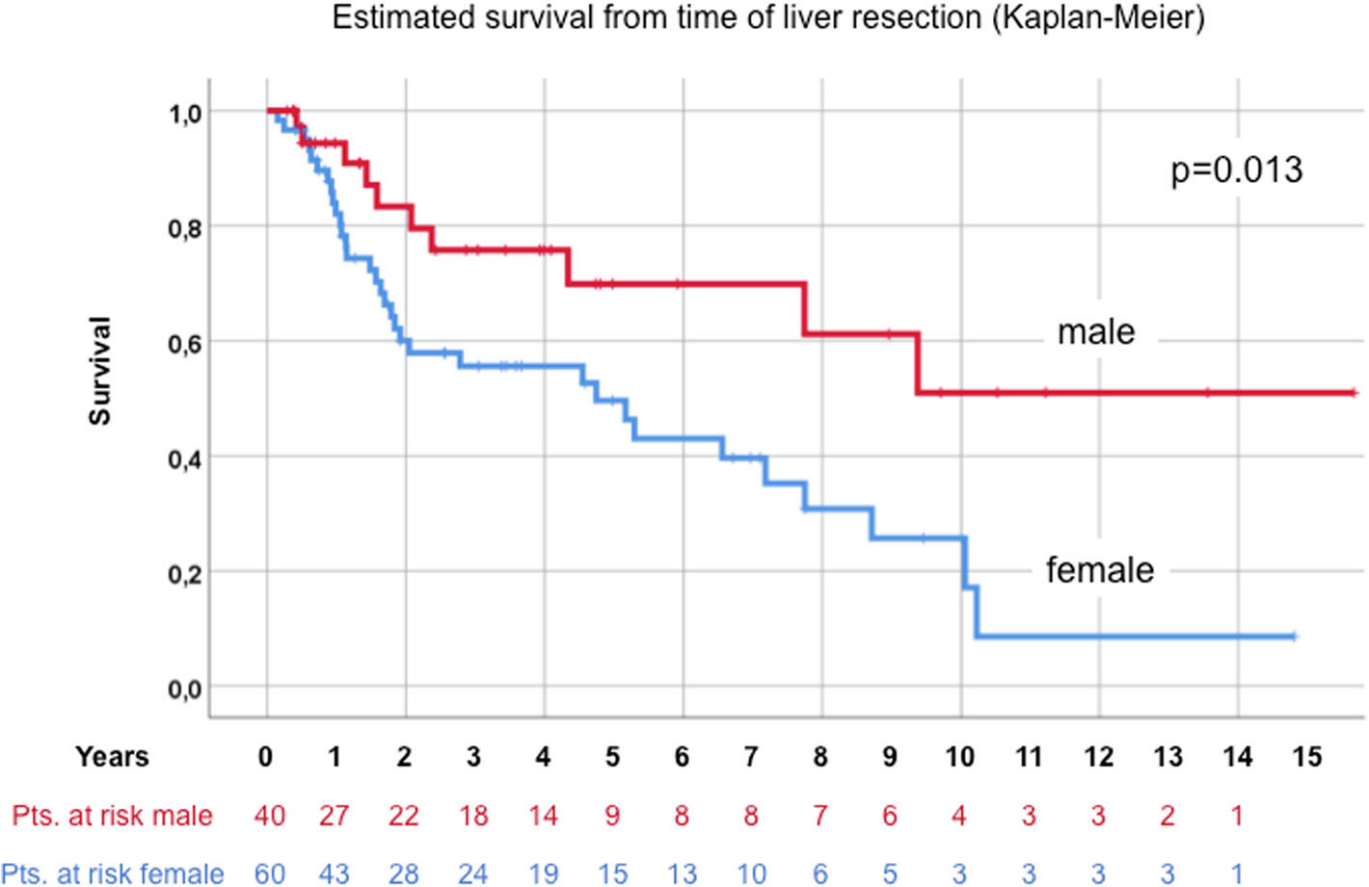
Male vs. Female Gender

In the entire group of 100 patients, overall survival at 5, 10 and 15 years after liver resection for NCNNE-LM was 56.8%, 34.3% and 25%, respectively (Fig. 2). Univariately, 10 patients with residual tumor (7 at the hepatic margin, 2 at the site of the primary tumor and 1 the hepatoduodenal ligament) showed a significantly lower estimated survival compared to 90 patients with no residual disease (*p* = 0.02, Fig. 3). Women were also shown to have poorer survival (25.7 vs. 51% at 10 years, *p* = 0.013, Fig. 4). There was no statistical difference between female gender specific tumors at 10 years (*n* = 30, e.g. breast, 30.6 vs. 37.2%) and others (*p* = 0.659).

Based on histology, there was no statistically-significant difference between adenocarcinomas, melanomas, GIST and sarcomas (*p* = 0.061). Nevertheless, GIST showed best 5- and 10-year estimated survival among all entities included in this series (90 and 77.1%, respectively).

Anatomical classification according to primary tumor origin by organ (e.g. gastrointestinal vs. genitourinary including subgroups, uveal vs. skin-derived melanoma) did not provide interpretable results in estimated survival differences (data not shown).

**Fig. 5:**
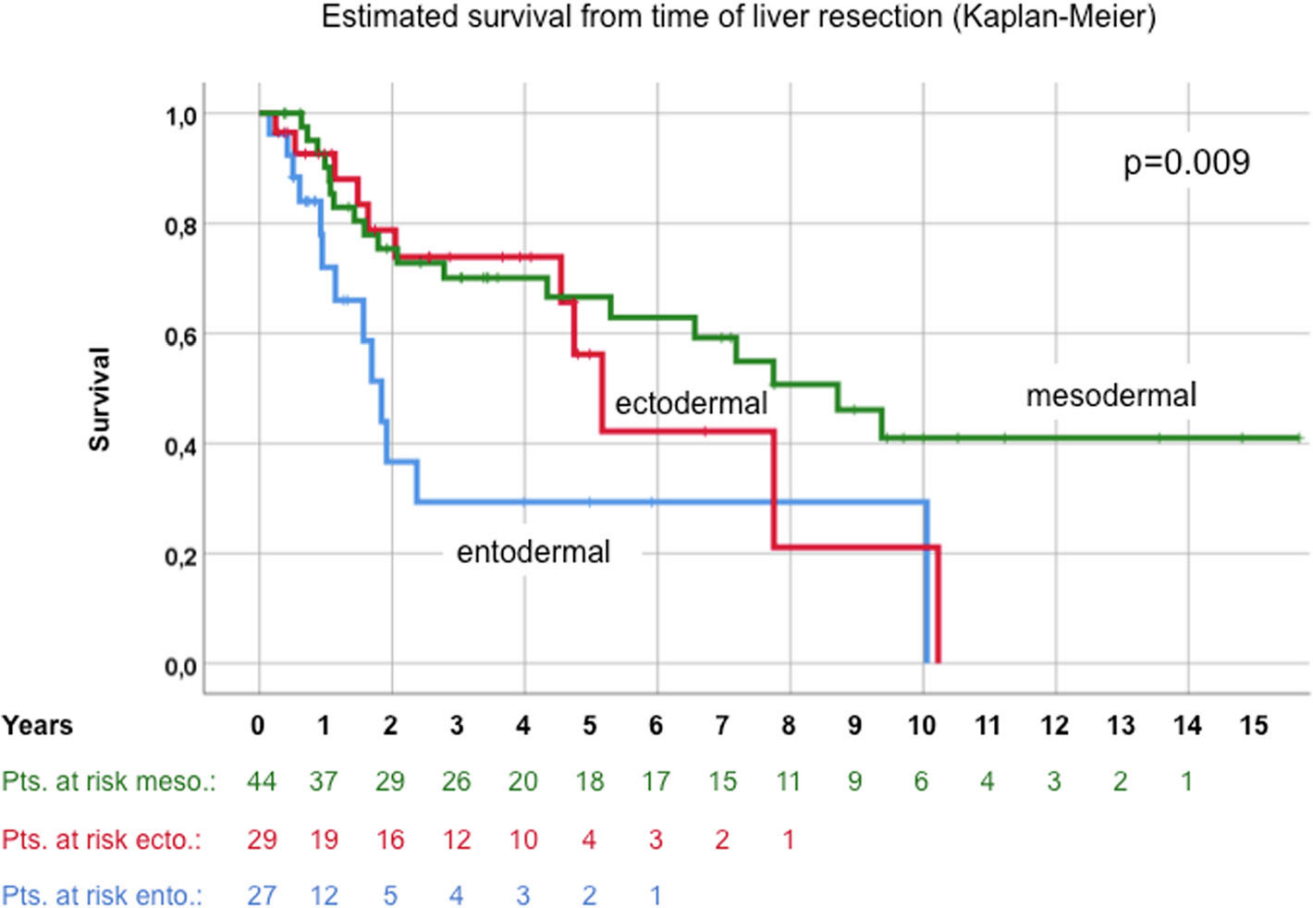
Embryologic Origin: meso- vs. ecto- vs. entodermal

Stratification by embryologic origin revealed best survival in patients with tumors arising from the mesoderm (66.6 and 41% estimated survival at 5 and 10 years, *p* = 0.009) compared to ecto- and entodermal tumor origin (Fig. 5).

**Fig. 6:**
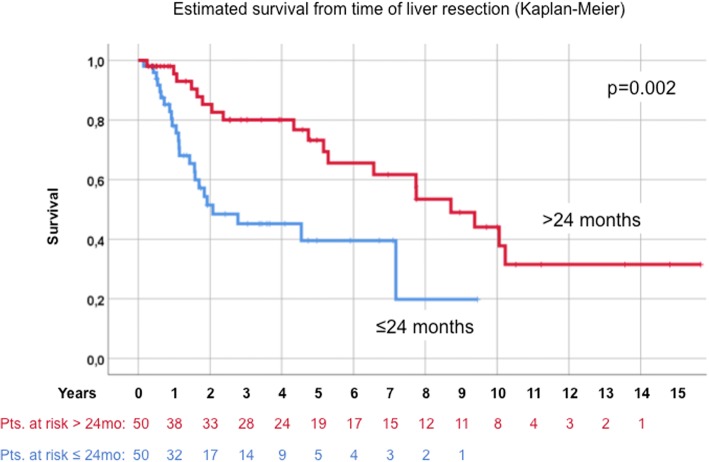
Onset of metastastic disease >24 vs. ≤24 months

Early onset metastatic disease as defined by diagnosis ≤24 months after primary tumor resection proved to be a strong prognostic factor (19.8 vs. 44.1% 10-year estimated survival compared to late onset metastatic disease (> 24 months, Fig. 6). The same was not true for synchronous (*n* = 20) or metachronous metastases (*n* = 80) if stratified by 6 months vs. later than 6 months (*p* = 0.147).

Other single factors from the Adam score (age, major liver resections and histology as shown above and for instance number and size of liver metastases resected did not significantly influence survival in our series (data not shown). A summary of the results is given in Table 2.

**Table 2:** Univariate analysis of estimated survival from time of liver resection (Kaplan-Meier)

Survival from time of liver resection				
Parameter	n	5-year	10-year	*p* value
Overall survival	100	56.8%	34.3%	–
Age				
> 60 years	49	62.10%	35.40%	0.669
≤ 60 years	51	52.10%	34.80%	
Gender specific tumor				
None female only	70	56.60%	37.20%	0.659
Female only	30	57.30%	30.60%	
Hepatic margin				
Negative	93	60%	34.40%	0.07
Positive	7	17.90%	17.90%	
Time of liver resection				
Interval resection	87	60.30%	35.10%	0.037
Simultaneous (with primary)	13	32.40%	32.40%	
Residual disease				
No residual tumor	90	60.70%	35.30%	0.02
Residual tumor	10	23.30%	23.30%	
Gender				
Male	40	69.90%	51%	0.013
Female	60	49.60%	25.70%	
Embryologic origin				
Mesodermal	44	66.60%	41%	
Ectodermal	29	56.30%	21.10%	0.009
Entodermal	27	29.30%	29.30%	
Onset of metastatic disease				
> 24 months	50	73.20%	44.10%	0.002
≤ 24 months	50	39.60%	19.80%	

### Multivariable survival analysis

**Fig. 7:**
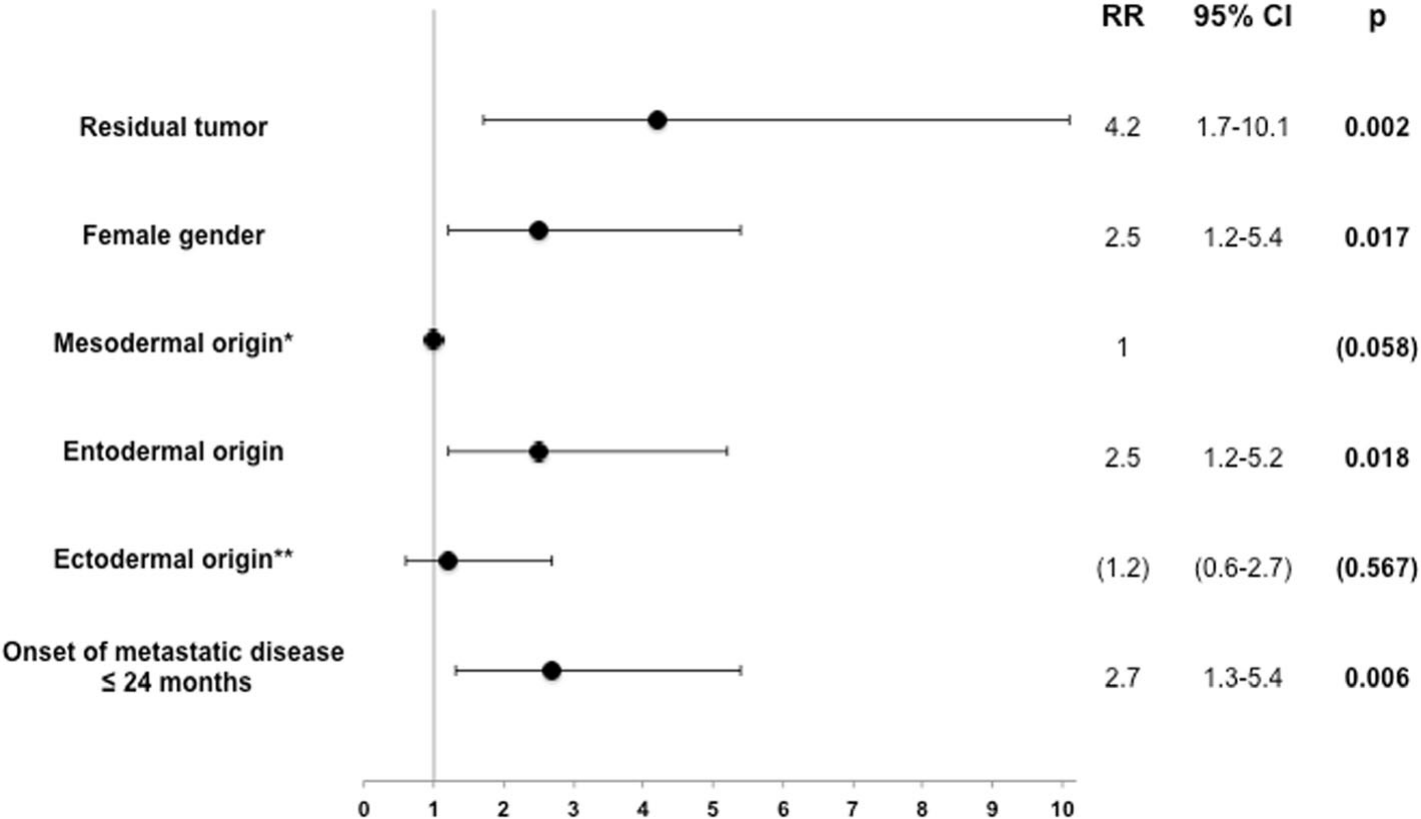
Multivariable Analysis

Multivariable (Cox) analysis of survival revealed that residual tumor was the strongest independent negative prognostic factor after resection of NCNNE LM (relative risk (RR) 4.2). Further independent negative prognostic factors were female gender (across all primary tumors) (RR 2.5), entodermal origin of the primary tumor (RR 2.5) compared to mesodermal origin, which has been set as the reference (RR 1) and early onset of metastatic disease ≤24 months (RR 2.7) (Fig. 7).

## Discussion

Today, only a few series exceeding 50 or 100 patients have been published shedding more light on future clinical decision-making by retrospectively analyzing outcomes [14–17, 19–24]. Adam et al. have published a paramount multicenter series in 2006, including 1452 patients with NCNNE from 1983 until 2004 [11]. Based on their collective, they have also developed a risk model in an attempt to predict long-time survival. Their score is based on the factors extrahepatic disease (EHD), major hepatectomy, incomplete (R2) resection, patient age, tumor histology and disease-free interval [11]. Hoffmann et al. demonstrated the applicability of this score in their own series [17]. Our study failed to reproduce those results presumably for two reasons. First, the Adam score was not applicable to the full extent here, because we have purposely excluded extrahepatic/extra-abdominal disease. Second, the proportion of tumor entities in the Adam series differs from our study population. Reason for this could be the rather large proportion of patients with melanoma as the primary tumor in our series (“worst” Adam score, 3 points, 14% vs. 7% Adam et al.) and the considerably smaller proportion of patients with breast cancer (“best” Adam score, 0 points,15% vs. 31% Adam et al.) [11]. Therefore, universal application of this score might be difficult and might indirectly correlate with the proportion of patients included in the inaugural paper. The group at the Memorial Sloan Kettering Cancer Center emphasize that hepatic resection in the setting of NCNNE clearly plays a role in multimodal therapy, but most likely is not per se a curative attempt [25]. The proportion of patients with EHD was around 30% in the studies cited by Tan et al., whereas again EHD in our series was specifically excluded. Hence, to strive for complete R0 resection as a “curative” attempt seems to be associated with prolonged survival in our series. Either way, Adam et al. estimate only 1–10% of patients with hepatic metastases from NCNNE tumors to be candidates for liver surgery [11].

This study focused on individual prognostic factors rather than including them in a score. With the exclusion of patients with extrahepatic or extra-abdominal disease, “curative” intent surgery was regarded as one mainstay of patient selection. This approach is supported by the result that residual disease has been shown to be the strongest predictive factor with an RR of 4.2 with regard to long-term survival. Even after adjusting for female specific tumors (30%, i.e. breast, ovarian, uterine, fallopian tube), female gender seems to have a negative prognostic impact in our series (RR 2.5). There is no conclusive explanation for this finding, especially after results from the largest series did not confirm this gender-specific difference on multivariable analysis. Therefore, we do not recommend precluding females or female gender- specific tumors from surgical therapy.

There has been a debate on how to define metachronous disease in NCNNE patients. Clearly, as shown by Adam et al., the “6 months rule” applied in CRC LM seems to fall short of being able to stratify patients with NCNNE. Metachronous (or late onset) metastatic disease heralds a potentially longer survival due to “slower” progression over time. Hoffmann et al. analyzed 150 patients using a cutoff of 24 months for “late onset” metastatic disease and were able to show metachronous disease to be associated with significantly longer survival on multivariate analysis [17]. It is of note that all survival data assessed in our study are calculated from the time of liver resection. We feel that this is particularly important in a “curative attempt” setting, because it takes into account that many patients with the diagnosis of NCNNE LM are not immediately referred to a surgeon. On the other hand, we could show that simultaneous resections of synchronous disease in select cases are not futile and can also yield long-term survival. It has to be stated that, on average, these patients are rare in our clinical setting. Probably owing to fact that synchronous LM from NCNNE are regarded as being “incurable” and therefore surgically “untreatable” by many teams. In conclusion, a “test of time” approach with a cutoff of 24 months to HR in NCNNE LM is probably helpful to select the best candidates, but not required per se for the indication of surgery.

Some insight into the highly varying results with regard to “tumor biology” might come from the comparison of the embryologic origin of the NCNNE LM primary tumor. This discussion has been fueled by findings addressing the right or left-sided location of the CRC primary tumor as an embryologically-driven potential key factor in survival [26]. Unfortunately, embryologic stratification alone does not eliminate overlap of groups with regard to e.g. the gender or the histologic classification of the primary. On multivariable analysis (with mesodermal origin defined as an RR of 1) entodermal origin was associated with poorer survival (RR 2.5). Ectodermal origin does seem to fall between the two other groups, and did not show any statistically- significant survival difference compared to either group. In summary, tumors from mesodermal origin, especially GIST, seem to have the most favorable long-term survival across a wide selection of published series [27, 28]. Therefore, some authors even advocate the exclusion of GIST from pooled NCNNE analyses in the future. These tumors have also been termed “non-sarcoma” liver metastases [16, 22].

There are several shortcomings in a retrospective study like this. Apart from clinically- based decision making in selecting patients over a time span of more than 15 years, there seem to be large differences between centers with regard to the proportion of patients sent for surgical evaluation and the timing of referral. Extrahepatic/extra-abdominal disease is also quite common in patients with NCNNE LM. Excluding them from analysis was done to achieve a homogeneous group. It does not reflect a policy to offer them no surgical treatment. We point out that at this point all negative predictive factors we found in this study are still not exclusion criterion for surgery at our center as long as “curative attempt surgery” can be planned.

## Conclusion

Hepatectomy for isolated NCNNE LM in well-selected patients is a feasible approach and results in a 5-year estimated survival after LR of 57% (including a 10-year and 15-year survival rate: 34% and 25%, respectively). Negative surgical margins, male gender, mesodermal origin of the primary and late onset metastatic disease (> 24 months) after therapy of the primary malignancy have proven to be robust positive prognostic factors in this study. In the absence of unanimously-reproducible established prognostic factors, individual decision-making in multidisciplinary discussion teams seems indispensable. Multicenter or prospective registry analysis of NCNNE-LM with regard to embryologic origin of the primary could provide more conclusive results, hopefully improving patient selection.

## Abbreviations

CRC: Colorectal carcinoma
EHD: Extrahepatic disease
GIST: Gastrointestinal stroma tumor
HR: Hepatic resection
LM: Liver metastases
mCRC: Metastatic colorectal carcinoma
NCNNE: Non-colorectal, non-neuroendocrine
NET: Neuroendocrine tumors
RR: Relative risk
SIRT: Selective internal radiotherapy
